# Modes of Brassinosteroid Activity in Cold Stress Tolerance

**DOI:** 10.3389/fpls.2020.583666

**Published:** 2020-11-06

**Authors:** Veronica E. Ramirez, Brigitte Poppenberger

**Affiliations:** Biotechnology of Horticultural Crops, School of Life Sciences Weihenstephan, Technical University of Munich, Freising, Germany

**Keywords:** steroid, resistance, frost, freezing, hormone, brassinosteroids, acclimation, abiotic stress

## Abstract

Cold stress is a significant environmental factor that negatively affects plant growth and development in particular when it occurs during the growth phase. Plants have evolved means to protect themselves from damage caused by chilling or freezing temperatures and some plant species, in particular those from temperate geographical zones, can increase their basal level of freezing tolerance in a process termed cold acclimation. Cold acclimation improves plant survival, but also represses growth, since it inhibits activity of the growth-promoting hormones gibberellins (GAs). In addition to GAs, the steroid hormones brassinosteroids (BRs) also take part in growth promotion and cold stress signaling; however, in contrast to Gas, BRs can improve cold stress tolerance with fewer trade-offs in terms of growth and yields. Here we summarize our current understanding of the roles of BRs in cold stress responses with a focus on freezing tolerance and cold acclimation pathways.

## Introduction

Cold stress represents a substantial risk for plant growth and development and impacts on plant distribution and crop production. Both chilling (>0°C) and freezing (<0°C) temperatures can cause damage, with the degree depending on the species and the developmental stage during exposure. Plants are most susceptible to frost during periods of active vegetative and reproductive growth, since growing, hydrated tissues are especially vulnerable to injury caused by freezing of cellular fluids (Nishiyama, 1995). Also at high risk is plant reproductive development, where both structural and functional abnormalities can lead to failed fruit and seed production (reviewed in Thakur et al., 2010; Albertos et al., 2019). Consequently, frost in spring during the bloom of fruit trees or stem elongation of winter cereal crops can result in a complete loss of harvest (Chmielewski et al., 2004; Augspurger, 2013).

**FIGURE 1:**
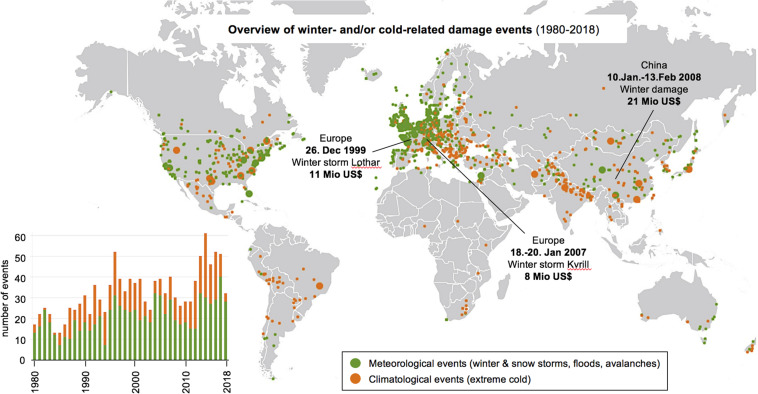
Summary of winter storm and winter damage events from 1980 to 2018. Data were retrieved from the public database provided by Munich RE NatCatSERVICE in July 2020. The world map shows the location where damage occurred with meteorological events in green and climatological events in orange. Highlighted are the three most costly single events in this time span, with the costs of damage in Mio US$. The bar chart on the left shows the number of worldwide events per year.

It is therefore perhaps not surprising that cold stress has significant economic impact. For example, the United States in the mid 20th century experienced more economic losses due to frost damage than to any other weather-related phenomenon (White and Haas, 1975). As a land spanning a range of climates, many of the southern subtropical and warm temperate latitudes are areas of horticultural and agricultural significance. Various fruit crops in these areas are vulnerable to frost, as physical damage to produce hinders ideal growth and reduces yield quality and quantity. Recent trends in winter-related damages also show an increasing number of incidents in the past century (Figure 1). In 2018, Harvey reported that up to 60% of locations across North America, Europe, East Asia, and parts of South America would see extreme weather events triple, much of which can be attributed to recent shifts in Arctic temperatures impacting the seasonal polar vortex.

Global warming is expected to further increase the risk of damage by frost in particular in mid-latitude zones where many major cropping areas lie. One such observation is the arctic amplification reduction of the pole–to–mid-latitude temperature gradient, predicted to cause more extreme weather events and extended conditions, such as early thaws, and sudden cold spells (Francis and Vavrus, 2012). The rising temperatures result in prolonged growing seasons, which delays cold hardening in fall and accelerates de-hardening in spring, increasing the potential for damage when early or late frosts occur (Rigby and Porporato, 2008; Augspurger, 2013; Hatfield and Prueger, 2015). Even in winter de-hardening could occur when temperatures rise periodically (Rapacz et al., 2017). Breeding for optimized cold stress tolerance may contribute to a possible solution, but has been mostly unsuccessful, as cold stress tolerance is usually correlated with impaired growth. This is supported widely by evidence that abiotic stress, including cold stress, represses growth, especially in scenarios of compounded stress or deprivation factors (Mittler, 2006). Growth repression is thought to free resources for other energy-demanding, stress-protective cellular reactions and systemic signaling (Mittler, 2002), although this hypothesis remains to be validated. Therefore, a thorough understanding of cold stress responses and how they are integrated with growth regulatory pathways is required for the design of targeted breeding approaches that aim to improve cold tolerance without trade-offs on growth or yield.

The repression of growth in response to cold involves effects on growth-promoting hormones and in this context, gibberellins (GAs) play an important role, although also other hormones are involved (reviewed in Eremina et al., 2016a). A reduction of GA levels and signaling activity contributes to restraint growth and enhances plant tolerance to several abiotic stress types including cold, drought, and osmotic stress (Achard et al., 2006, 2008; Magome et al., 2008). In addition to GAs, also the brassinosteroids (BRs) exhibit dual functions in growth control and abiotic stress protection. However, as opposed to GAs, there is evidence that BRs can promote both growth and resistance against certain abiotic stress types, which is intriguing since it may provide a means to increase abiotic stress tolerance with fewer trade-offs. A number of reviews have summarized progress in this area, providing an excellent overview of signaling events implicated in different abiotic stress types (reviewed in Divi and Krishna, 2009; Vriet et al., 2012; Planas-Riverola et al., 2019; Nolan et al., 2020). Here we have specifically focused on the role of BRs in cold stress responses and lay an emphasis on cold acclimation and freezing tolerance. While the resumption of growth after cold stress exposure is certainly also a relevant and exciting research area (reviewed in Vyse et al., 2019), evidence on the function of BRs in this process is still very limited (Pagter et al., 2017) and thus we do not expand on this topic here.

## Brassinosteroids and Their Role in Plant Growth and Frost Tolerance

Brassinosteroids are steroid hormones that are synthesized from the bulk sterol campesterol by multiple hydroxylation and oxidation events, which are catalyzed by different cytochrome P450 enzymes, including DWARF4 (DWF4), CONSTITUTIVE PHOTOMORPHOGENESIS AND DWARFISM (CPD), ROTUNDIFOLIA 3 (ROT3), and the CYP85A2 BR6ox2 (Clouse, 2011). The end products of BR biosynthesis castasterone (CS) and brassinolide (BL) are bioactive; they act in minute concentrations, which are closely monitored and adjusted. This is executed by regulation of genes involved in BR biosynthesis and catabolism, depending on the needs of intrinsic growth programs, but also according to requirements for growth adaptation and stress protection (reviewed in Clouse, 2011; Lv and Li, 2020). With respect to cold stress, there is evidence that the BR biosynthetic genes *DWF4*, *CPD*, and *BR6ox2* are rapidly downregulated by cold treatment (Eremina et al., 2016b). In particular, *BR6ox2* is repressed by one order of magnitude in plants exposed to 4°C, which is significant given that BR responses are usually only in the range of two to fourfold (Goda et al., 2002). Whether this repression of BR-biosynthetic genes also impacts BR levels and is of importance for BR-enabled effects in cold stress protection remains to be shown.

Castasterone and BL confer their bioactivity by binding to BR receptors of the BRASSINOSTEROID INSENSITIVE 1 (BRI1)-type, which initiates a phosphorylation-dependent signal transduction cascade that requires co-receptors, including BRI1-ASSOCIATED RECEPTOR KINASE 1 (BAK1) and BRI1-KINASE INHIBITOR 1 (BKI1), multiple phosphatases, including BSU1, and kinases, the most-studied being the GSK3/shaggy-like kinase BRASSINOSTEROID INSENSITIVE 2 (BIN2). BIN2 is a central repressor of BR signaling and, among other targets, also directly regulates BR-regulated transcription factors (TFs) that control BR-responsive gene expression (Kim and Wang, 2010). These TFs include different classical bHLH proteins such as BRASSINOSTEROID ENHANCED EXPRESSION 1-3 (BEE1-BEE3) and CESTA (CES), the BES1-INTERACTIVE MYC-LIKE (BIMs), and PHYTOCHROME INTERACTING FACTOR 4 (PIF4), but also atypical bHLH-type proteins, most importantly the BRI1-EMS-SUPPRESSOR 1/BRASSINAZOLE RESISTANT 1 (BES1/BZR1) subfamily (Wang et al., 2002; Yin et al., 2005; Bernardo-García et al., 2014; Khan et al., 2014).

Clear evidence for BRs being essential for plant development is the severe phenotypes of BR-deficient mutants. The most prominent features are dwarf growth with dark-green, cabbage-like leaves in the light, de-etiolated development in the dark, late flowering, and impaired fertility (reviewed in Clouse, 2011). Some of these defects are caused by malfunctioning cross-talk with GAs, since in certain plant species, including the model plant *Arabidopsis thaliana* and rice, BRs can promote GA biosynthesis (Tong et al., 2014; Unterholzner et al., 2015) and also interplay with GAs at the signaling level (reviewed in Tong and Chu, 2016; Unterholzner et al., 2016).

Severe growth defects represent a challenge when stress phenotypes are to be studied, since strong morphological alterations can impact stress perception. Therefore, multiple mutant settings and BR application studies were applied when the impact of BRs on freezing tolerance was assessed. This yielded solid evidence that BRs can improve frost tolerance. On the one hand, BR application increased survival rates of plants exposed to subzero temperatures (Kagale et al., 2007; Kim et al., 2010). On the other hand, and more importantly, *A. thaliana* mutants, deficient in various steps of BR signaling including the strong *bri1-1* and the weak *bri1-301* allele, and over-expression lines of *BIN2* and its homolog *ASKtheta*, showed decreased frost tolerance. In line, the *BRI1* over-expression line *35S:BRI1-GFP* and the higher-order *bin2-3 bil1 bil2* mutant, plants with constitutively active BR signaling, were more resistant to frost damage (Kim et al., 2010; Eremina et al., 2016b; Li et al., 2017). Interestingly, in addition to improving basal tolerance, BRs also contribute to acquired freezing tolerance in *A. thaliana*, which involves complex molecular and biochemical changes that are induced by low, but non-freezing temperatures, in a process termed cold acclimation.

## Biochemical and Cellular Events During Cold Stress

Cold acclimation enables plants from temperate geographical zones to increase their basal levels of freezing tolerance through initiation of a multitude of biochemical and cellular changes. These changes are induced by cold, but non-freezing temperatures and include compositional changes to the cell wall and membrane, an activation of anti-oxidative mechanisms, and the synthesis and accumulation of cryoprotective solutes, amino acids, and proteins (Lissarre et al., 2014).

**FIGURE 2:**
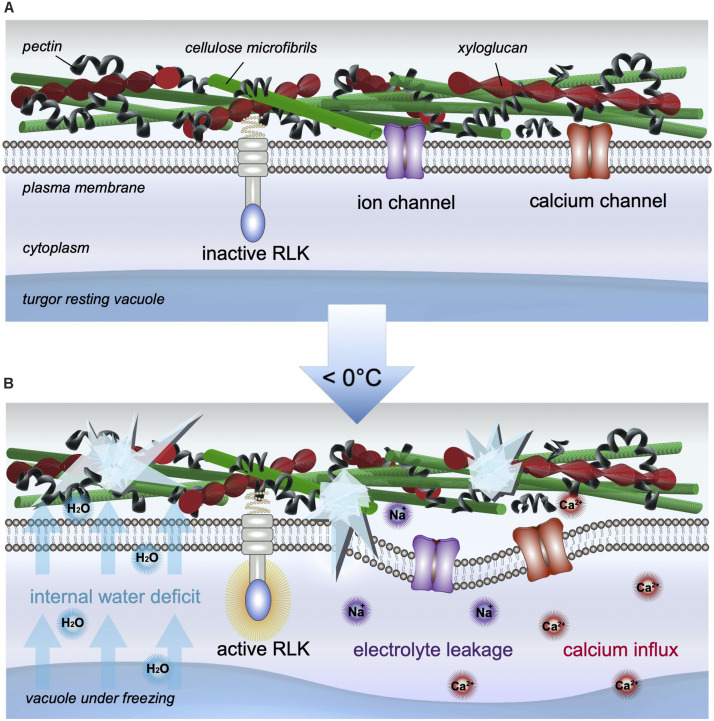
Cellular changes induced by freezing stress. **(A)** At ambient temperatures, the cell wall and membrane, as well as other cellular compartments, are intact and the cell is in a normal resting state, which ensures water and cellular volume homeostasis. **(B)** In the event of freezing stress, ice formation in the apoplast induces a movement of water out of the cell, which results in an internal water deficit, mimicking dehydration. If exposure is prolonged, intracellular ice may form, rupturing the plasma membrane, causing activation of RLKs, electrolyte leakage, calcium influx, and cell death, when the membrane eventually pulls away from the cell wall.

Frost damage can be caused by the freezing of soil waters leading to drought exposure, and by the freezing of cellular fluids, equally as problematic. The development of ice depends on the presence of ice nucleation sites, which may be intrinsically found in cells or cell walls, or formed by epiphytic bacteria found on leaves (Lindow et al., 1982). Although the formation of ice crystals is usually initiated in the cell walls and the intercellular space, it is the cellular water-deficit caused by both the lower chemical potential and vapor pressure of ice that actively dehydrates the cell. Water in the cytoplasm or vacuole moves down the potential water gradient toward extracellular ice, and across the plasma membrane (Buchanan et al., 2000). Freezing damage therefore induces cellular desiccation and rigidification of the cell membrane as it contracts and pulls away from the cell wall (Taiz et al., 2015; illustrated in Figure 2). Similar to the effects of drought stress, the symplast can lose about 90% of osmotically active water to the apoplast, putting already semi-dehydrated cells at risk for additional injury, in particular to further suffer membrane damage, which results in the loss of physical integrity of the cells (Taiz et al., 2015). On affected plants, wilting and water-soaked areas appear; when dried these form necrotic lesions on leaves, fruits, or stems (Morel and Dangl, 1997). While one obvious consequence may include the depreciation of aesthetic worth, the nutritional value, longevity, and overall yield may ultimately be compromised.

Frost itself is not a solitary threat; temperatures above zero 0°C can be problematic, especially for species that stem from tropical and subtropical geographical zones. Chilling can induce disruption of photosynthetic pathways, which activates photochemical production of reactive oxygen species (ROS) that cause injury to DNA, proteins, and lipids (Gururani et al., 2015). To prevent harm, plants utilize ROS scavenging enzymes and antioxidants such as ascorbate and glutathione (Mittler et al., 2004). In addition, denaturation of proteins, loss of guard cell function, and increased tissue levels of CO_2_ are observed (Allen and Ort, 2001). While chilling damage is evidently not attributed to ice crystal formation, it impacts on the plants ability to accommodate low temperatures with loss of membrane fluidity, resulting in membrane leakage (Verslues et al., 2006) and it appears that BRs can protect from the damaging effects of these events. BR application improved plant performance in the cold, which was shown for the chilling-sensitive species *Cucumis sativus* (cucumber), *Solanum lycopersicum* (tomato), *Oryza sativa* (rice), *Zea mays* (maize), and also for the cold-hardy plant *A. thaliana* (He et al., 1991; Katsumi, 1991; Kagale et al., 2007; Koh et al., 2007; Xia et al., 2009; Aghdam et al., 2012). Moreover, over-expression of *DWF4* conferred protective effects during germination and early seedling development of *A. thaliana* at 4°C (Divi and Krishna, 2009) and reduced chilling-induced oxidative damages in tomato (Xia et al., 2018). As a majority of high-value horticultural crops fall under the chilling-sensitive category, the function and efficacy of BR application demands a thorough understanding as to how each phase of injury may be transmitted, and this will be detailed here.

### Physical and Structural Changes During Cold Stress: The Cell Wall

The primary cell wall consists of a hemicellulose, pectin, and a structural protein matrix, amid an integrated network of cellulose microfibrils. In a normalized metabolic state, the proteins act as reinforcing structural components, operating together similar to an exoskeleton layer, resisting forces of turgor pressure, and controlling cell expansion (Taiz et al., 2015). The cell wall not only works as a diffusion barrier for ions and macromolecules, but it limits the range of molecules that can reach the plasma membrane by selective permeability and hydrophobic interactions, maintained by an intrinsic negative charge. In the event of perceived chilling stress, the membrane becomes rigid as protein conformations change and complexes destabilize. The level of susceptibility to cold-dependent conformational changes also depends on the cell type; typically, thicker cells walls are found in sclerenchyma cells (epidermal cells, xylem, phloem, and tracheids). The type and function of a plant cell also influences whether it is composed of a primary wall or secondary wall, which vary in development and protein and polysaccharide profiles.

Cell expansion and compression resistance rely on the abundance of pectin, which is found at relatively high levels in primary cell walls. Alternately, secondary cell walls contain dense structures mostly of cellulose-hemicellulose and lignin. These polysaccharide components determine stability, flexibility, and permeability; in the context of BRs in cold stress, evidence suggests BR signaling pathways to be involved with cell wall remodeling mechanisms responsible for altering these features (Rao and Dixon, 2017). In maize, wheat, and rice, for instance, the expression of many xyloglucan transferase/hydrolase enzymes (XTHs) and expansin genes was reported to be regulated by BRs (Uozu et al., 2000). XTHs catalyze cleavage of xylogulcan polymers then transferring ends to other xyloglucan chains, while expansins loosen linkages between cellulose microfibrils initiating cell wall loosening, a protective reaction against abiotic stresses (Yennawar et al., 2006). Such structural flexibility may allow a plant to survive harsh conditions with extreme humidity or temperature fluctuations.

Brassinosteroids have also been found to play an essential role in secondary cell wall formation. In *A. thaliana*, it was shown that BR signaling regulates secondary wall development via BR-induced BES1 activation of VND6 and VND7 (Yang and Wang, 2016), which determine xylem cell transition to form tracheary elements and alter the expression of MYB TFs that regulate lignin biosynthesis (Ohashi-Ito et al., 2010). Moreover, in *A. thaliana*, a loss of function of DIMINUTO1 (DIM1/DWF1), an enzyme that catalyzes an early step in BR biosynthesis, caused a phenotype with a significant reduction in lignin content and a lower lignin syringyl to guaiacyl ratio (Hossain et al., 2012). Lignin is the second most abundant carbon sink in plants; it is deposited in the secondary cell wall, augmenting cell wall rigidity and providing structural support, yet remaining clear of the primary cell wall (Karkonen and Koutaniemi, 2010); evidence that BRs can increase the accumulation of lignin has been implicated in the direct binding of BES1/BZR1 to promoter regions NAC and MYB TFs integral to lignin synthesis pathways (Benatti et al., 2012).

Alternately, pectin allows cell walls to remain firm, inhibiting collapse of the cellulose matrix, but also conferring flexibility, by forming hydrated gels, responsive to changes in polymer residues or pH (Voxeur and Hofte, 2016). In *A. thaliana*, BAK1 can interact with a plasma membrane receptor-like protein, RLP44, to repress pectin methylesterase inhibitor activity, reducing the rigidity of the pectic matrix and stimulating cell wall loosening in both basal and stressed conditions (Wolf et al., 2014). Although these implications of BR signaling in cell wall formation point to a significant contribution in cold stress tolerance, this area still requires further investigation.

In the event of cold exposure, both fluidity and strength of the cell wall influence internal cellular water conditions. Freezing especially alters the movement of water as intercellular ice forms. Ice formation occurs first in the apoplast where the negative water potential is far lower, eliciting further water movement down the gradient. Subsequently, an internal water deficit develops, mimicking the effects of cell dehydration (reviewed in Yamada et al., 2002; Le Gall et al., 2015). Moreover, freezing may induce intracellular ice crystal formation, and subsequently, wall destruction and cell death (reviewed in Tenhaken, 2015). As ice crystals grow, they puncture into the cytoplasm, rupturing membranes, and the membranes of nearby organelles. Both cellular desiccation and rigidification of the cell membrane continue as the membrane contracts and pulls away from the cell wall (Taiz et al., 2015). When the membrane then starts to break, an influx of calcium and electrolyte leakage occurs (illustrated in Figure 2), which is measurable and often serves as a quantifiable read-out for frost-induced damage.

### Membrane Fluidity

Exposure to cold or frost affects not only the permeability, flexibility, and resilience of the primary cell wall but if severe, may damage the plasma membrane. Low temperatures induce a hardening of the cell membrane, and a number of studies on cold perception suggest a dependency on membrane fluidity changes (Markovskaya and Shibaeva, 2017). As a major site of freezing-induced injury, the plasma membrane undergoes structural changes, a consequence of cellular dehydration (reviewed in Ingram and Bartels, 1996). When stages of cellular desiccation progress, the plasma membrane draws inward, away from the cell wall and closer to organelle membranes, both altering and destabilizing the integrity of membrane components, predominantly lipids and proteins (reviewed in Thomashow, 1999).

Under ambient growth conditions, each membrane in the plant cell has characteristic heterogeneous lipid profiles, and each class of lipids an equally distinct fatty acid composition. One such class of lipids are sterols, and include campesterol; as BR precursors, and central membrane components, they regulate membrane fluidity and permeability of membranes by directly affecting the activity of membrane-bound or membrane-associated proteins. As such they play a range of roles, from mature membrane protein signaling to inducing hyperpolarization of the membrane in cell division (reviewed by Clouse, 2011). Altered sterol profiles in BR mutants may affect membrane structure, influencing how specific signaling proteins interact, and impacting the fluidity of the membrane in response to environmental cues (Clouse, 2002). Although the current knowledge of membrane fluidity and BR signaling focuses on membrane-bound receptor activity and downstream signaling targets, other cell interactions like the ER-localized Unfolded Protein Response have been recently described to play a role both in protecting reproductive development stages from extreme temperatures and BR-mediated responses by recruiting the membrane-associated TFs bZIP17 and bZIP2 (Che et al., 2010; Bao and Howell, 2017). Rigidification of the membrane and alteration of lipid profiles may be resolute structural responses to cold exposure; however, further investigation of membrane-compartment interactions in vulnerable developmental stages may provide insight into otherwise cryptic BR-mediated signaling.

### Ca^2+^ Influx

A key molecular messenger prone to accumulation following cold perception is the divalent calcium cation Ca^2+^. In the cell, cytosolic calcium is normally maintained at low resting concentrations to facilitate external and internal Ca^2+^ membrane transport. Organelles including the rough ER and the vacuole contain intracellular stores of Ca^2+^ ready for signal-induced mobilization. The primary calcium receptor, calmodulin, is a highly conserved Ca^2+^ calcium binding protein attached to the plasma membrane, also found in both nuclear and cytoplasmic compartments. In the event of cold perception, a signal initiates channel-mediated, inward calcium transport. These channels are either activated mechanically by cell wall rigidification, or through direct ligand binding (Lissarre et al., 2014). It has been suggested that Ca^2+^ spiking is regulated by downstream receptor-like kinases with leucine-rich-repeat domains similar to those of the BR receptors (Oldroyd and Downie, 2004) and there is evidence that BRs can impact on the activity of Ca^2+^ channels (Straltsova et al., 2015); albeit, the modes are still unknown.

In *A. thaliana*, CaM (calmodulin) binds in a Ca^2+^-dependent manner to the cytoplasmic domain of BRI1 (Oh M. H. et al., 2012). Since BR signal transduction is initiated by hormone perception in the extracellular domain of BRI1, which then binds to BAK1, activating phosphorylation of cytoplasmic residues in the kinase domain by Ca^2+^/CaM binding may attenuate kinase activity of BRI1 and influence subsequent signaling and downstream target regulation (Du and Poovaiah, 2005; Oh et al., 2009).

Ca^2+^ binding to CaM has also been shown to be critical for BR biosynthesis and plant growth since it was found that DIM/DWF1 is a Ca^2+^/CaM-binding protein and that calmodulin-binding compromises DWF1 function *in planta* (Du and Poovaiah, 2005). DWF1 orthologs in other plant species have a similar Ca^2+^/CaM binding motif, indicating that Ca^2+^/CaM regulation of DWF1 and DWF1 homologs is conserved among plants (Du and Poovaiah, 2005). The possibility for a role of CaM in BR biosynthesis is also indicated by the fact that CaM over-expression lines show phenotypic features of plants over-expressing the BR biosynthetic gene *DWF4* (Du and Poovaiah, 2005). Consequently, the Ca^2+^/CaM complex may regulate a wide range of factors on the biosynthesis path of BRs in addition to LRR RLK co-receptor activity.

### ROS Species: Antioxidant Mobilization

Most reactions involving enzymatic kinetics interact with photosynthetic processes and metabolite accumulation to maintain a state of survival. ROS accumulate as a result of fewer scavenging enzymes and the disturbance of metabolic activity in response to abiotic stresses such as lowered temperatures or drought (Choudhury et al., 2017). Furthermore, over-reduction of the chloroplast electron chain may further increase ROS formation, leading to photoinhibition of PSI and PSII (Tjus et al., 2001). High concentrations of ROS lead to deterioration of membranes, causing membrane leakage of solutes, initiating a signal cascade responsive to the source of injury (Ruelland et al., 2009).

There is evidence that points to a BR function in the activation of cell-wall centered defense by ROS. In response to physical damage or pathogen inoculation, oxylipins, or oxygenated fatty acid products, might function as ROS signals to activate the BR pathway thereby reinforcing the cell wall defensively (Marcos et al., 2015). Furthermore, BRs may have an effect on bond integrity of monolignol polymers and phenolic acids in the cell wall by regulating antioxidant enzymes at both the transcriptional and post-transcriptional level (Li et al., 2016).

The exogenous application of BR increases the activity of antioxidant enzymes appreciably, including superoxide dismutase, catalase, ascorbate peroxidase, and peroxidase in grains exposed to high metal stress (reviewed in Kumar et al., 2015). This is thought to strengthen the mechanical properties of the wall by enhancing the covalent cross-linked components through combined peroxidase activity increase and ROS formation (Tenhaken, 2015). By utilizing the antioxidant defense system and facilitating cross-linking of phenolic compounds in the cell wall, BRs may orchestrate the alleviation of ROS-burst induced oxidative damage (Kumar et al., 2015).

Additionally, it has been reported that BRs play a role in the induced transcription of an NADPH oxidase-encoding gene, leading to increased levels of apoplastic H_2_O_2_. This rapid accumulation affects developmental and stress response activity by inducing biosynthesis of the plant hormone abscisic acid (ABA) and stomatal closure. By prolonging H_2_O_2_ accretion, it is suggested that BRs control ROS homeostasis to induce a level of plant stress tolerance (Xia et al., 2015). Moreover, BR-induced stimulation of antioxidant enzymes in response to high ROS levels appears to be relevant for ROS detoxification and thus plant survival following cold stress (Bajguz and Hayat, 2009; Liu et al., 2009).

### Biochemical and Physiological Changes Toward Acclimation

While many economically important crops are considered cold-sensitive, chilling-resistant plants such as *A. thaliana* are able to grow and develop even in low temperatures of 0–12°C, albeit at reduced rates. This adaptive capacity can be explained by diverse biochemical and physiological changes both in cell structure, and production of sugars, fatty acids, and secondary metabolites (Ruelland et al., 2009). In *A. thaliana*, various amino-acids including asparagine, aspartate, glutamate, glutamine, and alanine accumulate in response to cold (Klotke et al., 2004). Although a range of solutes accumulate, localization and therefore function vary within the cell. The trisaccharide raffinose, for instance, translocates from the cytosol to the chloroplasts, thereby protecting photosystems against damage in freeze-thaw phases (Knaupp et al., 2011). Conversely, in the plasma membrane, it behaves dispensably by replacing water molecules in the hydration shell of the lipid headgroups, preventing injurious lipid phase shifts.

Compositional changes in cell membrane lipid profiles are one of a series of physiological adjustments to cold conditions. In many cases, de-polymerization of the cytoskeleton in combination with phospholipid desaturation in the membrane can create an adaptive circumstance where the membrane rigidification is partially counterbalanced (Tasseva et al., 2004). Other responses to chilling include the plant increasing phospholipids or cerebrosides to prevent further membrane apposition and collapse.

While modifications improving structural integrity of the cell alleviate physical destruction, the accumulation of secondary metabolites may involve more complex pathways relying on gene regulation, expression, and modes of signal transduction (Shinozaki et al., 2003). Phenylpropanoids are a large group of secondary metabolites that comprise flavonoids, ubiquitous compounds involved in both abiotic and biotic stress defense mechanisms (Janská et al., 2010). Although well known for its role in fruit and leaf tissue pigmentation, UV protection, and photosynthetic interactions, very little is known regarding flavonoid pathway regulation in plants exposed to low temperatures, or its activity related to hormone signaling pathways, such as BRs. Petridis et al. (2016) described the change of a phenylpropanoid accumulation profile in BEE1 and GFR (*G2-LIKE FLAVONOID REGULATOR*) mutants following low temperature exposure, defined by quercetins and scopolin accumulating less, and anthocyanins accumulating more than in wild-type. These phenotypes formed the basis of further work, which showed that BEE1 and GFR act as negative regulators of anthocyanin accumulation by inhibiting anthocyanin biosynthesis genes, via suppression of the bHLH protein encoding genes *TRANSPARENT TESTA8* (*TT8*) and *GLABROUS3* (*GL3*) (Petridis et al., 2016). While BRs that act upstream of BEE1 have not been implicated into the complex rerouting of metabolic responses to low temperature, these data clearly imply BEE1 and potential redundantly acting factors as regulator(s), warranting further research in this field.

## Cold Stress Signaling Events

### *COR* Gene Regulation

In addition to the many changes that occur at the biochemical and morphological level, cold has a profound impact on the transcriptome; in *A. thaliana*, more than 2.500 genes are regulated by cold stress (Park et al., 2015). It can be assumed that the activation of cold responsive (*COR*) genes enables chilling stress protection and cold acclimation for increased freezing tolerance in capable plant species, although many of the described physiological events that take place when cold stress occurs have not been linked to up-stream signaling cascades as yet, and the sequence of events following cold stress exposure is often unclear.

*COR* gene regulation occurs in waves, is transient, and is realized by TFs. First wave TFs are activated at early time-points after cold perception and include ZAT10, ZAT12, HSFC1, CZF1, and ZF (Park et al., 2015). The by far best-characterized TFs that take part, however, are the C-REPEAT BINDING FACTORS (CBFs), also known as DROUGHT-RESPONSIVE ELEMENTS BINDING (DREB) factors, CBF1 (DREB1b), CBF2 (DREB1c), and CBF3 (DREB1a).

### BR Impact on the CBF Regulon

The CBFs are AP2/ERF family TFs that bind to the C-repeat (CRT)/dehydration-responsive element (DRE), a motif found in promoters of genes activated by cold stress, drought, or high salt exposure (Park et al., 2015). In *A. thaliana*, the CBF regulon of *COR* genes comprises 133 induced genes, including *COR15A*, *COR15B*, *COR47*, *COR78/RD29a*, *KIN1*, and *KIN2* which are often used as read-outs, and 39 repressed genes (Park et al., 2015). In *cbf1,2,3* triple mutants, which were generated using the CRISPR/Cas9 technology, basal freezing tolerance and cold acclimation are strongly compromised, providing conclusive evidence for the important role CBFs play in cold stress response (Zhao et al., 2016). Over-expression of *CBFs* increases freezing tolerance, as well as tolerance to drought and salinity in various plant species (Jaglo-Ottosen et al., 1998; Achard et al., 2008); however, it additionally causes dwarfism (Achard et al., 2008; Hu et al., 2015). This is attributed to the fact that CBFs promote expression of *GA2ox3 and GA2ox6*, which encode enzymes that inactivate GAs, and of *RGL3*, a DELLA protein that represses GA responses (Achard et al., 2008; Park et al., 2015). Since the expression of *CBFs* and a large set of CBF-induced genes is constitutively decreased in the BR-deficient mutants *bri1-1* and *bri1-301* (Eremina et al., 2016b), whereas it is constitutively increased in *BRI1* over-expressing plants (Kim et al., 2010; Eremina et al., 2016b) and the *bin2-3 bil1 bil2* triple mutant (Li et al., 2017), there is clear evidence that BRs are required for *CBF* transcription in basal, non-acclimated conditions.

**FIGURE 3:**
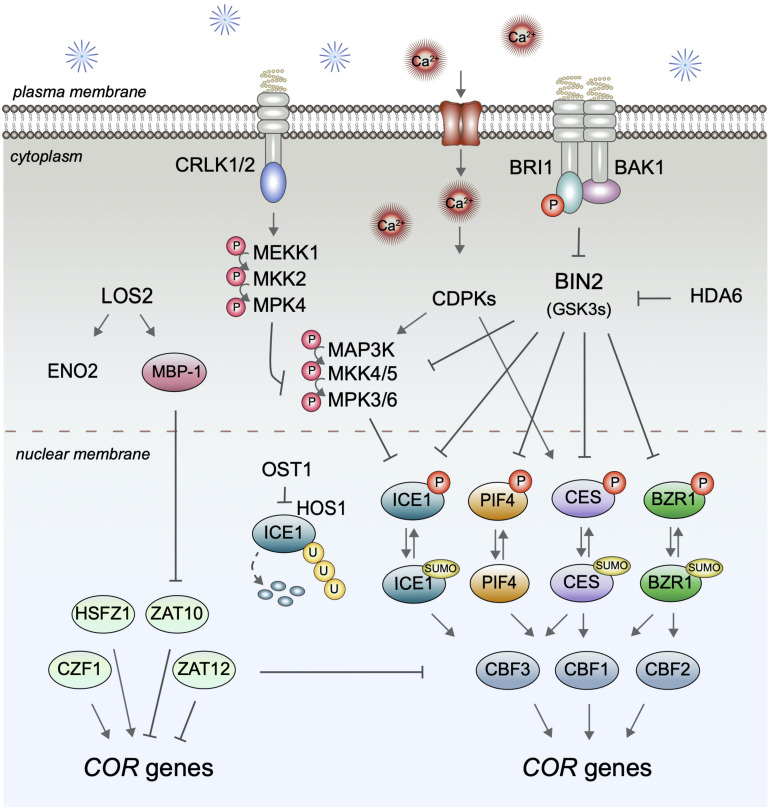
BR signaling and its impact on *CBF* expression. In the presence of BRs, BRI1 binds to BAK1, to activate inhibition of BIN2-type kinases and thereby impair the phosphorylation and inactivation of the transcription factors ICE1, PIF4, CES, and BZR1 that control expression of specific *CBFs*. Cold may impact this pathway by multiple means, including effects of calcium-activated CDPKs, CRLK1/2-controlled MAP kinases, and cold-induced SUMOylation events. In addition to *CBF*-control, BR signaling also impacts CBF-independent modes of *COR* gene regulation, our understanding of which is summarized on the left. However, how this activity is realized is yet unknown.

*CBF* expression is controlled by multiple upstream TFs (illustrated in Figure 3), the best-studied being the bHLH protein INDUCER OF CBF EXPRESSION 1/SCREAM 1 (ICE1/SCRM1) and its homolog ICE2/SCRM2, which control *CBF3* abundance (Ye et al., 2019). Importantly, recently it has been shown that ICE1 is a BIN2 target (Ye et al., 2019) linking it to the BR signaling pathway. BIN2 can directly phosphorylate ICE1, which reduces ICE1 protein stability and transcriptional activity, and is thought to allow for a repression of ICE1 in later stages of cold stress responses, when, following cold-induction, *CBF* expression requires a return to basal levels. In addition to BR signaling, BIN2 kinase activity is also controlled by acetylation catalyzed by the histone deacetylase HDA6, which is required for freezing tolerance (To et al., 2011; Hao et al., 2016), and thus HDA6 may contribute to a cold stress control of BIN2 activity with potential relevance for ICE1 and other BIN2-substrates.

In addition to ICE1, also the BR-regulated bHLH proteins CES and the BEEs take part in *CBF* control. CES can directly bind to the promoters of all three CBFs *in vitro* and *in vivo* and induce their expression (Eremina et al., 2016b). Since in *ces bee1 bee2 bee3* quadruple mutants the expression of *CBF1* and *CBF3*, but not of *CBF2*, was significantly repressed, it appears that the CES/BEE subfamily of bHLH proteins preferentially regulates *CBF1* and *CBF3* mRNAs (Eremina et al., 2016b), although redundancy in function with other bHLH proteins, or tissue-specific control, may have masked *CBF2* effects in the quadruple mutant. Higher order mutant combinations with additional bHLH proteins that target *CBFs* will be required to conclusively assess the relative contribution of the different bHLH proteins to *CBF* regulation. In this context, in addition to ICE1/2, also PIF4 and PIF7 should be considered, since both can control *CBF* expression (Lee and Thomashow, 2012), and PIF4 is a BIN2 target (Bernardo-García et al., 2014) that cooperates with BZR1 in transcriptional regulation (Oh E. et al., 2012).

ICE1 activity in CBF regulation is controlled by multiple upstream events with posttranslational modification being essential. The current postulation is that in response to cold stress ICE1 is phosphorylated by the ABA-regulated kinase OPEN STOMATA 1 (OST1). This inhibits ubiquitination by the UBQ E3 ligase HIGH EXPRESSION OF OSMOTICALLY RESPONSIVE GENES1 (HOS1) and degradation, resulting in ICE1 accumulation (Ding et al., 2015). Later, ICE1 is phosphorylated by the MAP kinases MPK3/6 (Zhao et al., 2017) and also by BIN2, which promotes HOS1 interaction (Ye et al., 2019), and, following ubiquitination, is degraded by the proteasome. MPK3/6 activities in cold stress responses are controlled by MKK5 and an additional unknown MAPK kinase, and adjusted by the MAPK kinase YDA and the MEKK1-MKK1/2-MPK4 cascade (Li et al., 2017; Zhao et al., 2017). YDA represses MPK3/6 activities in a cold-induced manner (Zhao et al., 2017), which may enable adjustment of *CBF* expression and repression of the pathway once *COR* genes have been activated and CBF removal is required (Ramirez and Poppenberger, 2017). In this context interestingly, BRs are known to impact on the YDA-MKK4/5-MPK3/6 MAP kinase module. BIN2 can phosphorylate both YDA (Kim et al., 2012) and MKK4/5 (Khan et al., 2013) and thereby repress MPK3/6 activities. As yet this cross-talk has only been shown to be relevant for stomatal patterning events (Wang and Estelle, 2014), however, clearly it will be important to assess, if it also contributes to BR function in *CBF* transcription and freezing tolerance.

In addition to phosphorylation and ubiquitination, SUMOylation also takes part in cold stress responses. Following cold exposure, the overall SUMOylation of proteins in plants drastically increases, and it has been shown that ICE1 SUMOylation enhances its activity in *CBF3* activation (Miura et al., 2007). Similarly, BZR1 and CES are SUMOylated (Khan et al., 2014; Srivastava et al., 2020) and there is evidence that SUMOylation promotes CES activity in freezing tolerance (Eremina et al., 2016b). While the upstream SUMO E3 ligase that targets CES has remained unknown, SIZ1 is a suitable candidate since SIZ1 SUMOylates ICE1 and, in addition to many other roles, is also important in freezing tolerance (Miura et al., 2007). How SUMOylation of ICE1 is induced is unclear, although a phosphorylation-deficient ICE1 mutant is more readily SUMOylated in response to cold (Miura et al., 2011). Phosphorylation also counteracts SUMOylation of CES, and there is some evidence that calcium-dependent protein kinases (CDPKs) are involved (Khan et al., 2014). CDPKs have both Ca^2+^ sensing and responding activities and can thereby directly translate Ca^2+^ signals into phosphorylation events. They are activated transiently to regulate TF activities either independently or in cross-talk with MAPK signaling cascades (Boudsocq and Sheen, 2013). CDPKs are thought to act as positive regulators of cold stress responses since cold induces expression of certain OsCDPKs and over-expression of the OsCDPK7 in rice, thereby increasing resistance to cold, drought, and salinity (Saijo et al., 2000). Whether CDPK-mediated phosphorylation of CES or other BR-regulated TFs contribute to *CBF* control remains to be addressed.

Another BR-regulated TF which functions in *CBF* regulation is BZR1. BZR1 can bind to the promoters of both *CBF1* and *CBF2* and in the dominant *bzr1-1D* mutant *CBF1* and *CBF2* mRNA levels and freezing tolerance are increased. Moreover, in response to cold stress accumulation of the de-phosphorylated, active form of BZR1 was promoted (Li et al., 2017). In addition to *CBF* regulation in basal growth conditions, there is evidence that BZR1 and the CES/BEE proteins contribute to CBF independent routes of *COR* gene regulation, which are much less studied today, but certainly worth a consideration.

### BRs in CBF-Independent Routes of *COR* Gene Regulation

The low temperature regulatory network beyond the CBF pathways is complex and highly interconnected and although it appears to account for the majority of cold stress responses in *A. thaliana* (Park et al., 2015), it has remained largely unstudied. One of the only CBF-independent signaling routes elucidated in some detail today is the control of *ZAT10* expression by LOS2, a bifunctional, cold-responsive locus that encodes both the TF MBP-1 and the enolase ENO2 (Lee et al., 2002; Kang et al., 2013; Eremina et al., 2015). However, neither up-stream events in LOS2 regulation by cold nor modes of ZAT10 activity in freezing tolerance are understood.

Brassinosteroids, in addition to promoting *CBF* expression in basal conditions, also take part in CBF-independent routes of *COR* gene activation and this likely accounts for the major part of their function in acquired freezing tolerance. A whole-transcriptome analysis of *bri1-301* plants following cold treatment identified non-CBF-regulon types of *COR* genes that depended on BRI1 activity. Importantly, there was a highly significant overlap of genes miss-regulated in both *bri1-301* and *ces bee1 bee2 bee3* quadruple mutant plants following cold exposure, providing evidence that the role of BRs in cold acclimation is conferred to a significant degree by the CES/BEE proteins (Eremina et al., 2016b). Among the *COR* genes that failed to be repressed in *bri1-301* and the *ces bee1 bee2 bee3* quadruple mutant, annotations involving cell cycle regulation, cell skeleton, and microtubule activity were overrepresented. This indicates that without a functional BR-CES/BEE module, plants are unable to decrease cell division and metabolism in the cold. Additionally, annotations for fatty acid and lipid synthesis, metabolism, and transport were over-represented (Eremina et al., 2016b) and it will therefore be interesting to see, if a role of CES/BEEs in these processes may contribute to their role in freezing tolerance.

Non-CBF-regulon targets of BZR1 include PYR1-LIKE 6 (PYL6), WRKY TF 6 (WRKY6), which play positive roles in ABA signaling, SENESCENCE-ASSOCIATED GENE 21 (SAG21), JASMONIC ACID CARBOXYL METHYLTRANSFERASE (JMT), and EPITHIOSPECIFIER MODIFIER1 (ESM1), which are involved in defense responses, and SUPPRESSOR OF OVEREXPRESSION OF CO 1 (SOC1), which is involved in flowering time control (Li et al., 2017). The relative contribution of CES/BEE and BZR1 down-stream targets to BR-conferred effects in freezing tolerance remains to be tested.

### BRs and Cold Stress Perception

While significant progress was made in elucidating signal transduction cascades that plants utilize to respond to cold stress, very little is still known about low temperature perception. A number of candidates for temperature sensors have been put forward and changes in membrane fluidity have been favored for some time. Membranes are the primary site of temperature perception. Their rigidification in response to cold will therefore likely impact on the activity of membrane-bound receptors, although transcriptome analysis showed no differences in *COR* gene expression between mutants that differed in membrane lipid saturation in *A. thaliana* (Knight and Knight, 2012).

Candidates of membrane-bound proteins that could serve as cold sensors are Histidine kinase Hik33, Bacillus subtilis histidine kinase DesK, and the mechanosensory Ca^2+^- and K^+^-channels (Markovskaya and Shibaeva, 2017). Since low temperature induces a transient influx of Ca^2+^ ions into the cytoplasm (Kiegle et al., 2000; Knight and Knight, 2001; Scrase-Field and Knight, 2003), it is thought that calcium channels serve as multifunctional sensors (Medvedev, 2005) that sense stress-induced changes in plasma membranes, including changing fluidity. In rice, COLD1 was proposed as a cold sensor, since it was found to be essential for chilling tolerance and play a role in the activation of Ca^2+^-channels in response to cold stress (Ma et al., 2015). Since there is evidence that BRs contribute to Ca^2+^influx by impacting on the activity of Ca^2+^-channels (Straltsova et al., 2015), it is conceivable that they could be taking part in cold perception modes, although this is another hypothesis that remains to be tested.

Another class of membrane-bound proteins, which likely contributes to the initiation of cold signaling, are calcium/calmodulin-regulated receptor-like kinases (CRLKs). In *A. thaliana*, CRLK1 and CRLK2 are required for the cold-induced induction of *CBFs* and act upstream of the MEKK1-MKK2-MPK4 module in the cold response pathway (Yang et al., 2010). Moreover, the COLD-RESPONSIVE PROTEIN KINASE 1 (CRPK1), which is activated by cold, was shown to phosphorylate 14-3-3 proteins that then shuttle from the cytoplasm to the nucleus to de-stabilize CBFs (Liu and Zhou, 2017). Since 14-3-3 proteins, through effects on BZR1 (Gampala et al., 2007) and BKI1 (Wang et al., 2011), are also involved in BR signaling in ambient conditions, it will be interesting to see if 14-3-3 proteins may also impact on BR activity in cold stress responses.

## Environmental Impact on BR Function in Cold Stress Responses

Temperature perception and signaling is strongly impacted by other environmental cues and also by intrinsic developmental programs. In particular, light is known to play an essential role in cold stress responses. Light is required for full cold acclimation (Wanner and Junttila, 1999) and induction of *CBF* expression (Kim et al., 2002; Soitama et al., 2008). A low red to far-red light ratio can increase *CBF* expression in a circadian-clock controlled manner even in the absence of cold, which is sufficient to improve freezing tolerance in *A. thaliana* (Franklin and Whitelam, 2007). In particular, the phytochrome light receptors PHYB and PHYD appear to be important in *CBF* regulation since in *phyB* and *pyhD* mutants the CBF-regulon is constitutively induced in *A. thaliana* (Franklin and Whitelam, 2007), tomato (Wang et al., 2016), and rice (He et al., 2017). Because PHYB regulates activity of PIF4 and PIF7, both of which are able to repress *CBF* expression in long-days (Lee and Thomashow, 2012), and PIF4 activity is also induced by BRs (Bernardo-García et al., 2014), there are indications for an interplay of light, cold, and BR signaling in *CBF* transcription. It remains to be tested, if this potential interplay may be mediated by a cooperation of BZR1 with PIF4, which as yet has only been shown to be relevant for growth control at ambient and high temperatures (Oh E. et al., 2012; Ibañez et al., 2018). Since light can also impact CBF-independent modes of cold stress responses (Lee et al., 2002), it is evident that much remains to be discovered about the complex cross-talk of light and cold stress responses in plants.

## Concluding Remarks

In the last few years, notable progress has been made in our understanding of the exceptional capacities of BRs to promote both growth and cold stress responses. It appears that in contrast to other growth-promoting hormones such as the GAs, BRs have the capability to uncouple tolerance to cold stress, and related abiotic stress types, from trade-offs in terms of growth and yield. This may be executed, at least in part, by the ability of BRs to promote *CBF* expression (Eremina et al., 2016b; Li et al., 2017) and to promote GA biosynthesis and signaling (Tong and Chu, 2016; Unterholzner et al., 2016) at the same time, which could release the repressive activity of CBFs on GA activity in growth induction. However, in addition to CBF-dependent effects, it is clear that BRs also act by CBF-independent modes. Moreover, in addition to controlling TF activities, BRs may also impact upstream events including cold perception and down-stream events in physiological responses and morphological adaptations. It will be exciting to discover how these effects are realized, and explore if they can be utilized for improvements in crop production when cold stress occurs.

## Author Contributions

VR and BP contributed equally to all aspects of this work. Both authors contributed to the article and approved the submitted version.

## Conflict of Interest

The authors declare that the research was conducted in the absence of any commercial or financial relationships that could be construed as a potential conflict of interest.

## References

[B1] Achard P. Cheng H. De Grauwe L. Decat J. Schoutteten H. Moritz T. (2006). Integration of plant responses to environmentally activated phytohormonal signals. *Science* 311 91–94. 10.1126/science.1118642 16400150

[B2] Achard P. Gong F. Cheminant S. Alioua M. Hedden P. Genschik P. (2008). The cold-inducible CBF1 factor–dependent signaling pathway modulates the accumulation of the growth-repressing DELLA proteins via its effect on gibberellin metabolism. *Plant Cell* 20 2117–2129. 10.1105/tpc.108.058941 18757556 PMC2553604

[B3] Aghdam M. S. Asghari M. Farmani B. Mohayeji M. Moradbeygi H. (2012). Impact of postharvest brassinosteroids treatment on PAL activity in tomato fruit in response to chilling stress. *Sci. Hortic.* 144 116–120. 10.1016/j.scienta.2012.07.008

[B4] Albertos P. Wagner K. Poppenberger B. (2019). Cold stress signalling in female reproductive tissues. *Plant Cell Environ.* 42 846–853. 10.1111/pce.13408 30043473

[B5] Allen D. J. Ort D. R. (2001). Impacts of chilling temperatures on photosynthesis in warm-climate plants. *Trends Plant Sci.* 6 36–42. 10.1016/S1360-1385(00)01808-2 11164376

[B6] Augspurger C. K. (2013). Reconstructing patterns of temperature, phenology, and frost damage over 124 years: spring damage risk is increasing. *Ecology* 94 41–50. 10.1890/12-0200.1 23600239

[B7] Bajguz A. Hayat S. (2009). Effects of brassinosteroids on the plant responses to environmental stresses. *Plant Physiol. Biochem.* 47 1–8. 10.1016/j.plaphy.2008.10.002 19010688

[B8] Bao Y. Howell S. H. (2017). The unfolded protein response supports plant development and defense as well as responses to abiotic stress. *Front. Plant Sci.* 8:344. 10.3389/fpls.2017.00344 28360918 PMC5350557

[B9] Benatti M. R. Penning B. W. Carpita N. C. McCann M. C. (2012). We are good to grow: dynamic integration of cell wall architecture with the machinery of growth. *Front. Plant Sci.* 3:187. 10.3389/Fpls.2012.00187 22936938 PMC3424494

[B10] Bernardo-García S. de Lucas M. Martínez C. Espinosa-Ruiz A. Davière J. M. Prat S. (2014). BR-dependent phosphorylation modulates PIF4 transcriptional activity and shapes diurnal hypocotyl growth. *Genes Dev.* 28 1681–1694. 10.1101/gad.243675.114 25085420 PMC4117943

[B11] Boudsocq M. Sheen J. (2013). CDPKs in immune and stress signaling. *Trends Plant Sci.* 18 30–40. 10.1016/j.tplants.2012.08.008 22974587 PMC3534830

[B12] Buchanan B. B. Gruissem W. Jones R. L. (2000). *Biochem. Mol Biol. Plants. 40.* Rockville, MD: American Society of Plant Physiologists 10.1023/A:1013849028622

[B13] Che P. Bussell J. D. Zhou W. Estavillo G. M. Pogson B. J. Smith S. M. (2010). Signaling from the endoplasmic reticulum activates brassinosteroid signaling and promotes acclimation to stress in *Arabidopsis*. *Sci. Signal.* 3:69. 10.1126/scisignal.2001140 20876872

[B14] Chmielewski F.-M. Müller A. Bruns E. (2004). Climate changes and trends in phenology of fruit trees and field crops in Germany, 1961–2000. *Agricult. Forest. Meterol.* 121 69–78. 10.1016/s0168-1923(03)00161-8

[B15] Choudhury F. K. Rivero R. M. Blumwald E. Mittler R. (2017). Reactive oxygen species, abiotic stress and stress combination. *Plant J.* 90 856–867. 10.1111/tpj.13299 27801967

[B16] Clouse S. D. (2002). *Arabidopsis* mutants reveal multiple roles for sterols in plant development. *Plant Cell* 14 1995–2000. 10.1105/tpc.140930 12215500 PMC543216

[B17] Clouse S. D. (2011). Brassinosteroid signal transduction: from receptor kinase activation to transcriptional networks regulating plant development. *Plant Cell* 23 1219–1230. 10.1105/tpc.111.084475 21505068 PMC3101532

[B18] Ding Y. Li H. Zhang X. Xie Q. Gong Z. Yang S. (2015). OST1 kinase modulates freezing tolerance by enhancing ICE1 stability in *Arabidopsis*. *Dev. Cell* 32 278–289. 10.1016/j.devcel.2014.12.023 25669882

[B19] Divi U. K. Krishna P. (2009). Brassinosteroid: a biotechnological target for enhancing crop yield and stress tolerance. *N. Biotechnol.* 26 131–136. 10.1016/j.nbt.2009.07.006 19631770

[B20] Du L. Poovaiah B. W. (2005). Ca 2+/calmodulin is critical for brassinosteroid biosynthesis and plant growth. *Nature* 437 741–745. 10.1038/nature03973 16193053

[B21] Eremina M. Rozhon W. Poppenberger B. (2016a). Hormonal control of cold stress responses in plants. *Cell. Mol. Life Sci.* 73 797–810. 10.1007/s00018-015-2089-6 26598281 PMC11108489

[B22] Eremina M. Rozhon W. Yang S. Poppenberger B. (2015). ENO 2 activity is required for the development and reproductive success of plants, and is feedback-repressed by A t MBP-1. *Plant J.* 81 895–906. 10.1111/tpj.12775 25620024

[B23] Eremina M. Unterholzner S. J. Rathnayake A. I. Castellanos M. Khan M. Kugler K. G. (2016b). Brassinosteroids participate in the control of basal and acquired freezing tolerance of plants. *Proc. Natl. Acad. Sci. U.S.A.* 113 E5982–E5991. 10.1073/pnas.1611477113 27655893 PMC5056081

[B24] Francis J. A. Vavrus S. J. (2012). Evidence linking Arctic amplification to extreme weather in mid-latitudes. *Geophys. Res. Lett.* 39:L06801 10.1029/2012GL051000

[B25] Franklin K. A. Whitelam G. C. (2007). Light-quality regulation of freezing tolerance in *Arabidopsis thaliana*. *Nat. Genet.* 39:1410. 10.1038/ng.2007.3 17965713

[B26] Gampala S. S. Kim T. W. He J. X. Tang W. Deng Z. Bai M. Y. (2007). An essential role for 14-3-3 proteins in brassinosteroid signal transduction in Arabidopsis. *Dev. Cell* 13 177–189. 10.1016/j.devcel.2007.06.009 17681130 PMC2000337

[B27] Goda H. Shimada Y. Asami T. Fujioka S. Yoshida S. (2002). Microarray analysis of brassinosteroid-regulated genes in *Arabidopsis*. *Plant Phys.* 130 1319–1334. 10.1104/pp.011254 12427998 PMC166652

[B28] Gururani M. A. Venkatesh J. Tran L. S. P. (2015). Regulation of photosynthesis during abiotic stress-induced photoinhibition. *Mol. Plant* 8 1304–1320. 10.1016/j.molp.2015.05.005 25997389

[B29] Hao Y. Wang H. Qiao S. Leng L. Wang X. (2016). Histone deacetylase HDA6 enhances brassinosteroid signaling by inhibiting the BIN2 kinase. *Proc. Natl. Acad. Sci. U.S.A.* 113 10418–10423. 10.1073/pnas.1521363113 27562168 PMC5027435

[B30] Hatfield J. L. Prueger J. H. (2015). Temperature extremes: effect on plant growth and development. *Weather. Clim. Extremes* 10 4–10. 10.1016/j.wace.2015.08.001

[B31] He R. Y. Wang G. J. Wang X. S. (1991). Effects of brassinolide on growth and chilling resistance of maize seedlings. *ACS Symposium Series* 474 220–230. 10.1021/bk-1991-0474.ch019

[B32] He X. L. Fan S. K. Zhu J. Guan M. Y. Liu X. X. Zhang Y. S. (2017). Iron supply prevents Cd uptake in *Arabidopsis* by inhibiting IRT1 expression and favoring competition between Fe and Cd uptake. *Plant. Soil* 416 453–462. 10.1007/s11104-017-3232-y

[B33] Hossain Z. McGarvey B. Amyot L. Gruber M. Jung J. Hannoufa A. (2012). *DIMINUTO 1* affects the lignin profile and secondary cell wall formation in *Arabidopsis*. *Planta* 235 485–498. 10.1007/s00425-011-1519-4 21947665

[B34] Hu Y. Wu Q. Sprague S. A. Park J. Oh M. Rajashekar C. B. (2015). Tomato expressing *Arabidopsis* glutaredoxin gene AtGRXS17 confers tolerance to chilling stress via modulating cold responsive components. *Hort. Res.* 2:15051. 10.1038/hortres.2015.51 26623076 PMC4641303

[B35] Ibañez C. Delker C. Martinez C. Bürstenbinder K. Janitza P. Lippmann R. (2018). Brassinosteroids dominate hormonal regulation of plant thermomorphogenesis via BZR1. *Curr. Biol.* 28 303–310. 10.1016/j.cub.2017.11.077 29337075

[B36] Ingram J. Bartels D. (1996). The molecular basis of dehydration tolerance in plants. *Annu. Rev. Plant Biol.* 47 377–403. 10.1146/annurev.arplant.47.1.377 15012294

[B37] Jaglo-Ottosen K. R. Gilmour S. J. Zarka D. G. Schabenberger O. Thomashow M. F. (1998). *Arabidopsis* CBF1 overexpression induces COR genes and enhances freezing tolerance. *Science* 280 104–106. 10.1126/science.280.5360.104 9525853

[B38] Janská A. Maršík P. Zelenková S. Ovesná J. (2010). Cold stress and acclimation–what is important for metabolic adjustment? *Plant Biol.* 12 395–405. 10.1111/j.1438-8677.2009.00299.x 20522175

[B39] Kagale S. Divi U. K. Krochko J. E. Keller W. A. Krishna P. (2007). Brassinosteroid confers tolerance in *Arabidopsis thaliana* and *Brassica napus* to a range of abiotic stresses. *Planta* 225 353–364. 10.1007/s00425-006-0361-6 16906434

[B40] Kang M. Abdelmageed H. Lee S. Reichert A. Mysore K. S. Allen R. D. (2013). At MBP-1, an alternative translation product of LOS 2, affects abscisic acid responses and is modulated by the E 3 ubiquitin ligase A t SAP 5. *Plant J.* 76 481–493. 10.1111/tpj.12312 23952686

[B41] Karkonen A. Koutaniemi S. (2010). Lignin biosynthesis studies in plant tissue cultures. *J. Integr. Plant Biol.* 52 176–185. 10.1111/j.1744-7909.2010.00913.x 20377679

[B42] Katsumi M. (1991). Physiological modes of brassinolide action in cucumber hypocotyl growth. *ACS Symposium Series* 474 246–254. 10.1021/bk-1991-0474.ch021

[B43] Khan M. Rozhon W. Bigeard J. Pflieger D. Husar S. Pitzschke A. (2013). Brassinosteroid-regulated GSK3/Shaggy-like kinases phosphorylate mitogen-activated protein (MAP) kinase kinases, which control stomata development in Arabidopsis thaliana. *J. Biol. Chem.* 288 7519–7527. 10.1074/jbc.M112.384453 23341468 PMC3597792

[B44] Khan M. Rozhon W. Unterholzner S. J. Chen T. Eremina M. Wurzinger B. (2014). Interplay between phosphorylation and SUMOylation events determines CESTA protein fate in brassinosteroid signalling. *Nat. Commun.* 5 1–10. 10.1038/ncomms5687 25134617 PMC4167607

[B45] Kiegle E. Moore C. A. Haseloff J. Tester M. A. Knight M. R. (2000). Cell-type-specific calcium responses to drought, salt and cold in the *Arabidopsis* root. *Plant J.* 23 267–278. 10.1046/j.1365-313x.2000.00786.x 10929120

[B46] Kim H. J. Kim Y. K. Park J. Y. Kim J. (2002). Light signalling mediated by phytochrome plays an important role in cold-induced gene expression through the C-repeat/dehydration responsive element (C/DRE) in *Arabidopsis thaliana*. *Plant J.* 29 693–704. 10.1046/j.1365-313X.2002.01249.x 12148528

[B47] Kim S. Y. Kim B. H. Lim C. J. Lim C. O. Nam K. H. (2010). Constitutive activation of stress-inducible genes in a brassinosteroid-insensitive 1 (bri1) mutant results in higher tolerance to cold. *Physiologia plantarum*, 138 191–204. 10.1111/j.1399-3054.2009.01304.x 20053182

[B48] Kim T. W. Michniewicz M. Bergmann D. C. Wang Z. Y. (2012). Brassinosteroid regulates stomatal development by GSK3-mediated inhibition of a MAPK pathway. *Nature* 482:419. 10.1038/nature10794 22307275 PMC3292258

[B49] Kim T. W. Wang Z. Y. (2010). Brassinosteroid signal transduction from receptor kinases to transcription factors. *Annu. Rev. Plant Biol.* 61 681–704. 10.1146/annurev.arplant.043008.092057 20192752

[B50] Klotke J. Kopka J. Gatzke N. Heyer A. G. (2004). Impact of soluble sugar concentrations on the acquisition of freezing tolerance in accessions of *Arabidopsis thaliana* with contrasting cold adaptation–evidence for a role of raffinose in cold acclimation. *Plant Cell. Environ.* 27 1395–1404. 10.1111/j.1365-3040.2004.01242.x

[B51] Knaupp M. Mishra K. B. Nedbal L. Heyer A. G. (2011). Evidence for a role of raffinose in stabilizing photosystem II during freeze–thaw cycles. *Planta* 234 477–486. 10.1007/s00425-011-1413-0 21533754

[B52] Knight H. Knight M. R. (2001). Abiotic stress signalling pathways: specificity and cross-talk. *Trends Plant Sci.* 6 262–267. 10.1016/S1360-1385(01)01946-X 11378468

[B53] Knight M. R. Knight H. (2012). Low-temperature perception leading to gene expression and cold tolerance in higher plants. *New Phytol.* 195 737–751. 10.1111/j.1469-8137.2012.04239.x 22816520

[B54] Koh S. Lee S. C. Kim M. K. Koh J. H. Lee S. An G. (2007). T-DNA tagged knockout mutation of rice OsGSK1, an orthologue of *Arabidopsis* BIN2, with enhanced tolerance to various abiotic stresses. *Plant Mol. Biol.* 65 453–466. 10.1007/s11103-007-9213-4 17690841

[B55] Kumar M. Campbell L. Turner S. (2015). Secondary cell walls: biosynthesis and manipulation. *J. Exp. Bot.* 67 515–531. 10.1093/jxb/erv533 26663392

[B56] Le Gall H. Philippe F. Domon J. M. Gillet F. Pelloux J. Rayon C. (2015). Cell wall metabolism in response to abiotic stress. *Plants* 4 112–166. 10.3390/plants4010112 27135320 PMC4844334

[B57] Lee C. M. Thomashow M. F. (2012). Photoperiodic regulation of the C-repeat binding factor (CBF) cold acclimation pathway and freezing tolerance in *Arabidopsis thaliana*. *Proc. Natl. Acad. Sci. U.S.A.* 109 15054–15059. 10.1073/pnas.1211295109 22927419 PMC3443188

[B58] Lee H. Guo Y. Ohta M. Xiong L. Stevenson B. Zhu J. K. (2002). LOS2, a genetic locus required for cold-responsive gene transcription encodes a bi-functional enolase. *EMBO J.* 21 2692–2702. 10.1093/emboj/21.11.2692 12032082 PMC126021

[B59] Li M. Ahammed G. J. Li C. Bao X. Yu J. Huang C. (2016). Brassinosteroid ameliorates zinc oxide nanoparticles-induced oxidative stress by improving antioxidant potential and redox homeostasis in tomato seedling. *Front. Plant Sci.* 7, 615. 10.3389/fpls.2016.00615 27242821 PMC4860460

[B60] Li C. Zhang S. Wang X. (2017). Novel signaling interface constituted with membrane receptor-like kinases emerged from the study of interaction and transphosphorylation of BRI1 and BAK1. *Curr. Top. Med. Chem.* 17 2393–2400. 10.2174/1568026617666170414144145 28413947

[B61] Lindow S. E. Arny D. C. Upper C. D. (1982). Bacterial ice nucleation: a factor in frost injury to plants. *Plant Physiol.* 70 1084–1089. 10.1104/pp.70.4.1084 16662618 PMC1065830

[B62] Lissarre M. Ohta M. Sato A. Miura K. (2014). Cold-responsive gene regulation during cold acclimation in plants. *Plant Signal. Behav.* 5 948–952. 10.4161/psb.5.8.12135 20699657 PMC3115169

[B63] Liu Y. Zhao Z. Si J. Di C. Han J. An L. (2009). Brassinosteroids alleviate chilling-induced oxidative damage by enhancing antioxidant defense system in suspension cultured cells of *Chorispora bungeana*. *Plant Growth Regul.* 59 207–214. 10.1007/s10725-009-9405-9

[B64] Liu Y. Zhou J. (2017). MAPping kinase regulation of ICE1 in freezing tolerance. *Trends Plant. Sci.* 23 91–93. 10.1016/j.tplants.2017.12.002 29248419

[B65] Lv M. Li J. (2020). Molecular mechanisms of brassinosteroid-mediated responses to changing environments in *Arabidopsis*. *Int. J. Mol. Sci.* 21 2737. 10.3390/ijms21082737 32326491 PMC7215551

[B66] Ma Y. Dai X. Xu Y. Luo W. Zheng X. Zeng D. (2015). COLD1 confers chilling tolerance in rice. *Cell* 160 1209–1221. 10.1016/j.cell.2015.01.046 25728666

[B67] Magome H. Yamaguchi S. Hanada A. Kamiya Y. Oda K. (2008). The DDF1 transcriptional activator upregulates expression of a gibberellin-deactivating gene, GA2ox7, under high-salinity stress in *Arabidopsis*. *Plant J.* 56 613–626. 10.1111/j.1365-313X.2008.03627.x 18643985

[B68] Marcos R. Izquierdo Y. Vellosillo T. Kulasekaran S. Cascón T. Hamberg M. (2015). 9-Lipoxygenase-derived oxylipins activate brassinosteroid signaling to promote cell wall-based defense and limit pathogen infection. *Plant Physiol.* 169 2324–2334. 10.1104/pp.15.00992 26417008 PMC4634075

[B69] Markovskaya E. F. Shibaeva T. G. (2017). Low temperature sensors in plants: hypotheses and assumptions. *Biol. Bull. Rev.* 44 150–158. 10.1134/S1062359017020145

[B70] Medvedev S. S. (2005). Calcium signaling system in plants. *Russ. J. Plant Physiol.* 52 249–270. 10.1007/s11183-005-0038-1

[B71] Mittler R. (2002). Oxidative stress, antioxidants and stress tolerance. *Trends Plant Sci.* 7 405–410. 10.1016/S1360-1385(02)02312-9 12234732

[B72] Mittler R. (2006). Abiotic stress, the field environment and stress combination. *Trends Plant Sci.* 11 15–19. 10.1016/j.tplants.2005.11.002 16359910

[B73] Mittler R. Vanderauwera S. Gollery M. Van Breusegem F. (2004). Reactive oxygen gene network of plants. *Trends Plant Sci.* 9 490–498. 10.1016/j.tplants.2004.08.009 15465684

[B74] Miura K. Jin J. B. Lee J. Yoo C. Y. Stirm V. Miura T. (2007). SIZ1-mediated sumoylation of ICE1 controls CBF3/DREB1A expression and freezing tolerance in *Arabidopsis*. *Plant Cell* 19 1403–1414. 10.1105/tpc.106.048397 17416732 PMC1913760

[B75] Miura K. Ohta M. Nakazawa M. Ono M. Hasegawa P. M. (2011). ICE1 Ser403 is necessary for protein stabilization and regulation of cold signaling and tolerance. *Plant J.* 67 269–279. 10.1111/j.1365-313X.2011.04589.x 21447070

[B76] Morel J. B. Dangl J. L. (1997). The hypersensitive response and the induction of cell death in plants. *Cell Death Differ.* 4:671. 10.1038/sj.cdd.4400309 16465279

[B77] Nishiyama I. (1995). Damage due to extreme temperatures. *Rice Sci.* 2 769–812.

[B78] Nolan T. M. Vukašinovć N. Liu D. Russinova E. Yin Y. (2020). Brassinosteroids: Multidimensional regulators of plant growth, development, and stress responses. *Plant Cell*, 32 295–318. 10.1105/tpc.19.00335 31776234 PMC7008487

[B79] Oh E. Zhu J. Y. Wang Z. Y. (2012). Interaction between BZR1 and PIF4 integrates brassinosteroid and environmental responses. *Nature Cell Biol.* 14:802. 10.1038/ncb2545 22820378 PMC3703456

[B80] Oh M. H. Kim H. S. Wu X. Clouse S. D. Zielinski R. E. Huber S. C. (2012). Calcium/calmodulin inhibition of the *Arabidopsis* BRASSINOSTEROID-INSENSITIVE 1 receptor kinase provides a possible link between calcium and brassinosteroid signalling. *Biochem. J.* 443 515–523. 10.1042/BJ20111871 22309147 PMC3316158

[B81] Oh M. H. Wang X. Kota U. Goshe M. B. Clouse S. D. Huber S. C. (2009). Tyrosine phosphorylation of the BRI1 receptor kinase emerges as a component of brassinosteroid signaling in *Arabidopsis*. *Proc. Natl. Acad. Sci. U.S.A.* 106 658–663. 10.1073/pnas.0810249106 19124768 PMC2613937

[B82] Ohashi-Ito K. Oda Y. Fukuda H. (2010). *Arabidopsis* VASCULAR-RELATED NAC-DOMAIN6 directly regulates the genes that govern programmed cell death and secondary wall formation during xylem differentiation. *Plant Cell* 22 3461–3473. 10.1105/tpc.110.075036 20952636 PMC2990123

[B83] Oldroyd G. E. Downie J. A. (2004). Calcium, kinases and nodulation signalling in legumes. *Nat. Rev. Mol. Cell. Biol.* 5 566–576. 10.1038/nrm1424 15232574

[B84] Pagter M. Alpers J. Erban A. Kopka J. Zuther E. Hincha D. K. (2017). Rapid transcriptional and metabolic regulation of the deacclimation process in cold acclimated Arabidopsis thaliana. *BMC Genomics*, 18 731. 10.1186/s12864-017-4126-3 28915789 PMC5602955

[B85] Park S. Lee C. M. Doherty C. J. Gilmour S. J. Kim Y. Thomashow M. F. (2015). Regulation of the *Arabidopsis* CBF regulon by a complex low-temperature regulatory network. *Plant J.* 82 193–207. 10.1111/tpj.12796 25736223

[B86] Petridis A. Döll S. Nichelmann L. Bilger W. Mock H. P. (2016). Arabidopsis thaliana G2-LIKE FLAVONOID REGULATOR and BRASSINOSTEROID ENHANCED EXPRESSION1 are low-temperature regulators of flavonoid accumulation. *New Phytol.* 211 912–925. 10.1111/nph.13986 27125220

[B87] Planas-Riverola A. Gupta A. Betegón-Putze I. Bosch N. Ibañes M. Caño-Delgado A. I. (2019). Brassinosteroid signaling in plant development and adaptation to stress. *Development*, 146. 10.1242/dev.151894 30872266 PMC6432667

[B88] Ramirez V. E. Poppenberger B. (2017). MAP kinase signaling turns to ICE. *Dev. Cell* 43 545–546. 10.1016/j.devcel.2017.10.032 29207256

[B89] Rao X. Dixon R. A. (2017). Brassinosteroid mediated cell wall remodeling in grasses under abiotic stress. *Front. Plant Sci.* 8:806. 10.3389/fpls.2017.00806 28567047 PMC5434148

[B90] Rapacz M. Jurczyk B. Sasal M. (2017). Deacclimation may be crucial for winter survival of cereals under warming climate. *Plant Sci.* 256 5–15. 10.1016/j.plantsci.2016.11.007 28167038

[B91] Rigby J. R. Porporato A. (2008). Spring frost risk in a changing climate. *Geophys. Res. Let.* 35:L12703 10.1029/2008gl033955

[B92] Ruelland E. Vaultier M. N. Zachowski A. Hurry V. (2009). Cold signalling and cold acclimation in plants. *Adv. Bot. Res.* 49 35–150. 10.1016/S0065-2296(08)00602-2

[B93] Saijo Y. Hata S. Kyozuka J. Shimamoto K. Izui K. (2000). Over-expression of a single Ca2+-dependent protein kinase confers both cold and salt/drought tolerance on rice plants. *Plant J.* 23 319–327. 10.1046/j.1365-313x.2000.00787.x 10929125

[B94] Scrase-Field S. A. Knight M. R. (2003). Calcium: just a chemical switch? *Curr. Opin. Plant Biol.* 6 500–506. 10.1016/S1369-5266(03)00091-8 12972052

[B95] Shinozaki K. Yamaguchi-Shinozaki K. Seki M. (2003). Regulatory network of gene expression in the drought and cold stress responses. *Curr. Opin. Plant Biol.* 6 410–417. 10.1016/S1369-5266(03)00092-X 12972040

[B96] Soitama A. J. Piippo M. Allahverdiyea Y. Battchikova N. Aro E. M. (2008). Light has a specific role in modulating *Arabidopsis* gene expression at low temperature. *BMC Plant Biol.* 8:13. 10.1186/1471-2229-8-13 18230142 PMC2253524

[B97] Srivastava M. Srivastava A. K. Orosa-Puente B. Campanaro A. Zhang C. Sadanandom A. (2020). SUMO conjugation to BZR1 enables brassinosteroid signaling to integrate environmental cues to shape plant growth. *Curr. Biol.* 30:1423.e3. 10.1016/j.cub.2020.01.089 32109396 PMC7181186

[B98] Straltsova D. Chykun P. Subramaniam S. Sosan A. Kolbanov D. Sokolik A. (2015). Cation channels are involved in brassinosteroid signalling in higher plants. *Steroids* 97 98–106. 10.1016/j.steroids.2014.10.008 25449770

[B99] Taiz L. Zeiger E. Moller I. M. Murphy A. (2015). *Plant Physiology and Development*, 6th Edn Sunderland, CT: Sinauer Associates.

[B100] Tasseva G. Richard L. Zachowski A. (2004). Regulation of phosphatidylcholine biosynthesis under salt stress involves choline kinases in *Arabidopsis thaliana*. *FEBS Lett.* 566 115–120. 10.1016/j.febslet.2004.04.015 15147879

[B101] Tenhaken R. (2015). Cell wall remodeling under abiotic stress. *Front. Plant Sci.* 5:771. 10.3389/fpls.2014.00771 25709610 PMC4285730

[B102] Thakur P. Kumar S. Malik J. A. Berger J. D. Nayyar H. (2010). Cold stress effects on reproductive development in grain crops: an overview. *Environ. Exp. Bot.* 67 429–443. 10.1016/j.envexpbot.2009.09.004

[B103] Thomashow M. F. (1999). Plant cold acclimation: freezing tolerance genes and regulatory mechanisms. *Annu. Rev. Plant Biol.* 50 571–599. 10.1146/annurev.arplant.50.1.571 15012220

[B104] Tjus S. E. Scheller H. V. Andersson B. Møller B. L. (2001). Active oxygen produced during selective excitation of photosystem I is damaging not only to photosystem I, but also to photosystem II. *Plant Physiol.* 125 2007–2015. 10.1104/pp.125.4.2007 11299380 PMC88856

[B105] To T. K. Nakaminami K. Kim J. M. Morosawa T. Ishida J. Tanaka M. (2011). *Arabidopsis* HDA6 is required for freezing tolerance. *Biochem. Biophys. Res. Commun.* 406 414–419. 10.1016/j.bbrc.2011.02.058 21329671

[B106] Tong H. Chu C. (2016). Reply: brassinosteroid regulates gibberellin synthesis to promote cell elongation in rice: critical comments on Ross and Quittenden’s letter. *Plant Cell* 28 833–835. 10.1105/tpc.16.00123 27006487 PMC4863391

[B107] Tong H. Xiao Y. Liu D. Gao S. Liu L. Yin Y. (2014). Brassinosteroid regulates cell elongation by modulating gibberellin metabolism in rice. *Plant Cell* 26 4376–4393. 10.1105/tpc.114.132092 25371548 PMC4277228

[B108] Unterholzner S. J. Rozhon W. Papacek M. Ciomas J. Lange T. Kugler K. G. (2015). Brassinosteroids are master regulators of gibberellin biosynthesis in *Arabidopsis*. *Plant Cell* 27 2261–2272. 10.1105/tpc.15.00433 26243314 PMC4568508

[B109] Unterholzner S. J. Rozhon W. Poppenberger B. (2016). Reply: interaction between Brassinosteroids and Gibberellins: synthesis or signaling? In *Arabidopsis*, Both! *Plant Cell* 28 836–839. 10.1105/tpc.16.00120 27006486 PMC4863389

[B110] Uozu S. Tanaka-Ueguchi M. Kitano H. Hattori K. Matsuoka M. (2000). Characterization of XET-related genes of rice. *Plant Physiol.* 122 853–859. 10.1104/Pp.122.3.853 10712549 PMC58921

[B111] Verslues P. E. Agarwal M. Katiyar-Agarwal S. Zhu J. Zhu J. K. (2006). Methods and concepts in quantifying resistance to drought, salt and freezing, abiotic stresses that affect plant water status. *Plant J.* 45 523–539. 10.1111/j.1365-313X.2005.02593.x 16441347

[B112] Voxeur A. Hofte H. (2016). Cell wall integrity signaling in plants: “To grow or not to grow that’s the question”. *Glycobiology* 26 950–960. 10.1093/glycob/cww029 26945038

[B113] Vriet C. Russinova E. Reuzeau C. (2012). Boosting crop yields with plant steroids. *Plant Cell* 24 842–857. 10.1105/tpc.111.094912 22438020 PMC3336137

[B114] Vyse K. Pagter M. Zuther E. Hincha D. K. (2019). Deacclimation after cold acclimation—a crucial, but widely neglected part of plant winter survival. *J. Exp. Bot.* 70 4595–4604. 10.1093/jxb/erz229 31087096 PMC6760304

[B115] Wang H. Wang H. Shao H. Tang X. (2016). Recent advances in utilizing transcription factors to improve plant abiotic stress tolerance by transgenic technology. *Front. Plant Sci.* 7:67. 10.3389/fpls.2016.00067 26904044 PMC4746321

[B116] Wang H. Yang C. Zhang C. Wang N. Lu D. Wang J. (2011). Dual role of BKI1 and 14-3-3 s in brassinosteroid signaling to link receptor with transcription factors. *Dev. Cell* 21 825–834. 10.1016/j.devcel.2011.08.018 22075146

[B117] Wang R. Estelle M. (2014). Diversity and specificity: auxin perception and signaling through the TIR1/AFB pathway. *Curr. Opin. Plant. Biol.* 21 51–58. 10.1016/j.pbi.2014.06.006 25032902 PMC4294414

[B118] Wang Z. Y. Nakano T. Gendron J. He J. Chen M. Vafeados D. (2002). Nuclear-localized BZR1 mediates brassinosteroid-induced growth and feedback suppression of brassinosteroid biosynthesis. *Dev. Cell* 2 505–513. 10.1016/s1534-5807(02)00153-3 11970900

[B119] Wanner L. A. Junttila O. (1999). Cold-induced freezing tolerance in *Arabidopsis*. *Plant Physiol.* 120 391–400. 10.1104/pp.120.2.391 10364390 PMC59277

[B120] White G. F. Haas J. E. (1975). *Assessment of Research on Natural Hazards.* Cambridge, MA: MIT Press.

[B121] Wolf S. Van Der Does D. Ladwig F. Sticht C. Kolbeck A. Schürholz A. K. (2014). A receptor-like protein mediates the response to pectin modification by activating brassinosteroid signaling. *Proc. Natl. Acad. Sci. U.S.A.* 111 15261–15266. 10.1073/pnas.1322979111 25288746 PMC4210321

[B122] Xia X. J. Fang P. P. Guo X. Qian X. J. Zhou J. Shi K. (2018). Brassinosteroid-mediated apoplastic H2O2-glutaredoxin 12/14 cascade regulates antioxidant capacity in response to chilling in tomato. *Plant Cell Environ.* 41 1052–1064. 10.1111/pce.13052 28776692

[B123] Xia X. J. Wang Y. J. Zhou Y. H. Tao Y. Mao W. H. Shi K. (2009). Reactive oxygen species are involved in brassinosteroid-induced stress tolerance in cucumber. *Plant Physiol.* 150 801–814. 10.1104/pp.109.138230 19386805 PMC2689980

[B124] Xia X. J. Zhou Y. H. Shi K. Zhou J. Foyer C. H. Yu J. Q. (2015). Interplay between reactive oxygen species and hormones in the control of plant development and stress tolerance. *J. Exp. Bot.* 66 2839–2856. 10.1093/jxb/erv089 25788732

[B125] Yamada T. Kuroda K. Jitsuyama Y. Takezawa D. Arakawa K. Fujikawa S. (2002). Roles of the plasma membrane and the cell wall in the responses of plant cells to freezing. *Planta* 215 770–778. 10.1007/s00425-002-0814-5 12244442

[B126] Yang J. H. Wang H. (2016). Molecular mechanisms for vascular development and secondary cell wall formation. *Front. Plant Sci.* 7:356. 10.3389/fpls.2016.00356 27047525 PMC4801872

[B127] Yang T. Shad Ali G. Yang L. Du L. Reddy A. S. N. Poovaiah B. W. (2010). Calcium/calmodulin-regulated receptor-like kinase CRLK1 interacts with MEKK1 in plants. *Plant Signal. Behav.* 5 991–994. 10.4161/psb.5.8.12225 20724845 PMC3115177

[B128] Ye K. Li H. Ding Y. Shi Y. Song C. Gong Z. (2019). BRASSINOSTEROID-INSENSITIVE2 negatively regulates the stability of transcription factor ICE1 in response to cold stress in *Arabidopsis*. *Plant Cell* 31 2682–2696. 10.1105/tpc.19.00058 31409630 PMC6881119

[B129] Yennawar N. H. Li L. C. Dudzinski D. M. Tabuchi A. Cosgrove D. J. (2006). Crystal structure and activities of EXPB1 (Zea m 1), alpha, beta-expansin and group-1 pollen allergen from maize. *Proc. Natl. Acad. Sci. U.S.A.* 103 14664–14671. 10.1073/pnas.0605979103 16984999 PMC1595409

[B130] Yin Y. Vafeados D. Tao Y. Yoshida S. Asami T. Chory J. (2005). A new class of transcription factors mediates brassinosteroid-regulated gene expression in *Arabidopsis*. *Cell* 120 249–259. 10.1016/j.cell.2004.11.044 15680330

[B131] Zhao C. Wang P. Si T. Hsu C. C. Wang L. Zayed O. (2017). MAP kinase cascades regulate the cold response by modulating ICE1 protein stability. *Dev. Cell* 43 618–629. 10.1016/j.devcel.2017.09.024 29056551 PMC5716877

[B132] Zhao C. Zhang Z. Xie S. Si T. Li Y. Zhu J. K. (2016). Mutational evidence for the critical role of CBF Transcription Factors in cold acclimation in *Arabidopsis*. *Plant Physiol*. 171:00533. 10.1104/pp.16.00533 27252305 PMC4972280

